# Culturally Competent Practice: A Mixed Methods Study Among Students, Academics and Alumni of Clinical Psychology Master’s Programs in the Netherlands

**DOI:** 10.5964/ejop.v14i1.1461

**Published:** 2018-03-12

**Authors:** Lennie R. C. Geerlings, Claire L. Thompson, Vivian Kraaij, Ger P. J. Keijsers

**Affiliations:** a Institute of Cultural Anthropology and Development Sociology, Leiden University, Leiden, the Netherlands; b College of Arts, Society and Education, James Cook University, Townsville, Australia; c School of Psychology, The Cairnmillar Institute, Melbourne, Australia; d Department of Psychology, James Cook University, Townsville, Australia; e Department of Psychology, Leiden University, Leiden, the Netherlands; f Behavioural Science Institute, Radboud University, Nijmegen, the Netherlands; g Clinical Psychological Science, Maastricht University, Maastricht, the Netherlands; Webster University Geneva, Geneva, Switzerland; The Maria Grzegorzewska University, Warsaw, Poland

**Keywords:** cultural competence, clinical psychology education, Multicultural Awareness, Knowledge and Skills Survey (MAKSS), Interpretative Phenomenological Analysis (IPA), Netherlands

## Abstract

This is the first research into preparation for multicultural clinical psychology practice in Europe. It applies the theory of multicultural counselling competency (MCC) to a case study in the Netherlands. It was hypothesized that cross-cultural practice experience, identification as a cultural minority, and satisfaction with cultural training was associated with MCC. The Multicultural Awareness Knowledge and Skills Survey was completed by 106 participants (22 students, 10 academics, 74 alumni) from clinical psychology masters’ programs. MANOVA detected a main effect of cross-cultural experience on MCC for all groups and universities. The data were enriched with exploratory qualitative data from 14 interviews (5 students, 5 academics, 4 alumni). Interpretative Phenomenological Analysis revealed three themes: limitations of clinical psychology, strategies for culturally competent practice, and strategies for cultural competency development. These outcomes suggest that cultural competency continues to require attention in master’s programs. The paper makes recommendations for further research enquiry related to training clinical psychologists to practice in Europe’s multicultural societies.

In 2008, an advisory report to the Ministry of Health in the Netherlands recommended research into the effectiveness of competency profiles for cross-cultural practice (Bekker & van Mens-Verhulst, 2008, p. 31). In the preceding years, a growing body of literature had been developed on skills for culturally sensitive practice (e.g., Knipscheer & Kleber, 2005; Kramer, 2004; Kramer & Sbiti, 2007), in the context of sustained critique on the mental health care sector for excluding cultural minorities (Knipscheer, Drogendijk, & Mooren, 2011; Van Dijk, 2008). Cultural minorities, often referred to as *allochtonen* in the Netherlands, currently make up about 20% of the Dutch population (Centraal Bureau voor de Statistiek, 2014). They are former migrants or refugees from Turkey, Morocco, Suriname, the (former) Netherlands Antilles, Eastern Europe or other regions of the world, or have one or both parents with such a background (Centraal Bureau voor de Statistiek & Sociaal Cultureel Planbureau, 2013). The cultural diversity is likely to further increase as a result of large numbers of people from the Middle East and Northern Africa, among other regions of the world, seeking refuge in the Netherlands (Vluchtelingenwerk, 2015). It is thus of paramount importance that clinical psychologists are prepared to work cross-culturally. However, since the 2008 call for action, no research has been conducted on cultural competency for clinical psychologists in the Netherlands.

The present study aims to address this research gap. It focuses on preparation in universities for culturally competent clinical psychology practice. In the Netherlands, clinical psychology postgraduate master’s degrees are the first professional clinical psychology training experience for future practitioners. Master’s graduates work as therapists under supervision of more experienced clinical psychologists (called *GZ psycholoog*). Two additional years of training and supervised practice at a tertiary institution are required to work independently. However, these post-master training places are highly restricted, thus the master is frequently the last opportunity to prepare for multicultural practice. Thus, it is pertinent to research how future psychologists are prepared for culturally competent practice in clinical psychology master’s education. This issue is explored with a theoretical focus on a standardised model of cultural competency.

The model of *Multicultural Counselling Competency* (MCC; Sue et al., 1982) may potentially inform cultural competency training in the Netherlands (Kramer & Sbiti, 2007). The tripartite model of MCC asserts that culturally competent psychologists need to develop *awareness*, *knowledge* and *skills* (Sue et al., 1982). Multicultural awareness refers to practitioners’ consciousness of their own biases, beliefs and values, and how these may affect clients. Multicultural knowledge refers to familiarity with cultural groups and social and political systems in practitioners’ own country and the client’s country of origin. Finally, multicultural skills refer to the ability to communicate adequately and appropriately with a variety of cultural groups, and to exercise adequate intervention skills on behalf of culturally diverse clients (Sue et al., 1982). Although the MCC model has been altered (e.g., Campinha-Bacote, 1994; Sodowsky, Taffe, Gutkin, & Wise, 1994; Sue, Arredondo, & McDavis, 1992), the original 1982 MCC model remains most influential at research and policy levels. It is thus a logical starting point for exploring cultural competency training.

Research evidence suggests that psychologists with higher levels of MCC may be more effective in cross-cultural practice. Client outcomes are improved (Worthington, Soth-McNett, & Moreno, 2007), possibly through the practitioners’ enhanced cultural knowledge and self-awareness (Sammons & Speight, 2008), cultural empathy (Burkard & Knox, 2004; Chao, Wei, Good, & Flores, 2011), and decreased racial prejudice (Castillo, Brossart, Reyes, Conoley, & Phoummarath, 2007; Chao et al., 2011). In the Netherlands, cultural training informed by the MCC model was positively appreciated by psychology professionals and students (Kramer & Sbiti, 2007). This suggests it is worthwhile to further assess the effectiveness of the MCC model in guiding cultural competency development.

There may be several pathways in which psychologists develop MCC. American evidence shows that multicultural training is associated with self-reported MCC (D’Andrea, Daniels, & Heck, 1991), especially when students were satisfied with the cultural training they received (Tummala-Narra, Singer, Li, Esposito, & Ash, 2012). In addition to training, MCC also developed with cultural exposure: psychology students or practitioners with more cross-cultural experience reported higher levels of cultural competency compared to their less experienced colleagues (Allison, Crawford, Echemendia, LaVome, & Knepp, 1994; Hansen et al., 2006; Sehgal et al., 2011). In addition, students who identified as belonging to a cultural minority reported higher levels of MCC compared to their peers (Pope-Davis, Reynolds, Dings, & Nielson, 1995). It needs to be assessed whether the possible pathways to cultural competency, including multicultural training, cross-cultural experience and belonging to a cultural minority, are applicable in a Dutch context.

Therefore, research explores two questions. Firstly: Are multicultural training, cross-cultural experience and belonging to a cultural minority associated with culturally competency of students, academics and alumni of clinical psychology master’s programs? This will be examined through a standardised self-assessment of MCC, the Multicultural Awareness, Knowledge and Skills Survey (MAKSS; D’Andrea et al., 1991). Research indicates social desirability as a possible covariate of self-reported MCC (Constantine & Ladany, 2000). Therefore, it was hypothesized that, after controlling for the effects of social desirability, satisfaction with cultural training cross-cultural practice experience, and personal identification as a cultural minority would be associated with students’, academics’ and alumni’s multicultural counselling competency (Study 1). As this is the first research using a standardised measure of MCC in Dutch universities, this study also seeks to supplement the quantitative MCC data with more exploratory interview data. The second research question is: How do students, academics, and alumni of clinical psychology experience preparation for culturally competent clinical psychology practice? This qualitative question is explored using interview data (Study 2).

## Study 1: Survey on Multicultural Counselling Competency

It was hypothesized that after controlling for the effects of social desirability:

H1: Satisfaction with cultural training was associated with students’, academics’ and alumni’s multicultural counselling competency.

H2: Cross-cultural practice experience was associated with students’, academics’ and alumni’s multicultural counselling competency.

H3: Personal identification as a cultural minority was associated with students’, academics’ and alumni’s multicultural counselling competency.

### Method

#### Participants

In total 132 participants responded to an online survey, however 26 participants responded to less than half of the questions and were excluded from the analysis, bringing the sample size to 106. The final sample consisted of 22 students of clinical psychology master’s programs (age: *M* = 25.09, *SD* = 3.77), 10 academics currently teaching into these programs (age: *M* = 43.00, *SD* = 10.71), and 74 alumni of these programs (age: *M* = 33.49, *SD* = 12.23). Demographic information of survey participants is detailed in Table 1.

**Table 1:** Questionnaire Participant Characteristics (n = 106)

Characteristic	Students (*n* = 22)		Academics (*n* = 10)		Alumni (*n* = 74)	
	*n*	%	*n*	%	*n*	%
Gender						
Male	2	9.1	4	40	13	17.6
Female	20	90.9	6	60	61	82.4
Nationality						
Dutch	16	72.7	10	100	62	82.2
Double^a^	0	–	0	–	1	.9
Other	6	27.3	0	–	12	16.8
Cultural identification						
Cultural majority Dutch^b^	15	68.2	10	100	60	80
Cultural minority Dutch^c^	0	–	0	–	4	5.3
Other	7	31.8	0	–	11	16.3
Cross-cultural experience						
Yes	3	13.6	0	–	14	18.7
No	19	86.4	10	100	61	81.3

^a^Double nationality which includes the Dutch nationality. ^b^Participants who identified as *autochtoon*. ^c^Cultural minority Dutch includes participants who identified as *allochtoon* or Netherlands Antilles.

Seventy participants were affiliated with University A and thirty-seven with University B. Both universities provided a one-year master’s program in clinical psychology, with a very similar curriculum, however, one university differentiated itself by its rigorous research training, while the other university embedded clinical psychology with the other fields of applied psychology. One university was located in a densely populated multicultural region, the other bordered Germany. Both universities attracted a relatively large number of international students; one university had a separate international master’s program taught in English.

#### Instruments

The online questionnaire included a demographic section, and assessments of satisfaction with cultural training, MCC, and social desirability. The survey was conducted in English, so no translation of the assessments was required, and international students, academics and alumni could participate. To assess readability and clarity, the questionnaire was piloted among one student and two alumni of a clinical psychology program at a university not otherwise involved in the study, and minor revisions took place based on the received feedback.

##### Demographics questionnaire

In line with previous studies on MCC (e.g., Lee & Khawaja, 2013; Pope-Davis et al., 1995) the demographics section included age, gender, university affiliation and role, cultural background and cultural training or work experiences.

##### Satisfaction with cultural training

Based on Tummala-Narra et al. (2012), four statements, such as “*Awareness, knowledge and clinical skills required for clinical psychology practice in multicultural Netherlands are adequately addressed in my clinical psychology program*”, could be responded to on a 7-point Likert scale ranging from *strongly disagree* to *strongly agree.* The sum of the item scores provided the measure of satisfaction with multicultural training with higher scores indicating greater satisfaction.

##### Multicultural Awareness Knowledge and Skills Survey (MAKSS)

The MAKSS (D’Andrea et al., 1991) consists of 60 questions or statements that could be answered on a 4-point Likert-scale, ranging from *very limited* or *strongly disagree* to *very good* or *strongly agree*. The three subscales reflect Sue et al.’s (1982) MCC: Multicultural Awareness (Items 1-20, e.g., *“At this point in your life, how would you rate your understanding of the impact of the way you think and act when interacting with persons of different cultural backgrounds?”*), Multicultural Knowledge (Items 21-40, e.g., *“What do you think of the following statement?: the difficulty with the concept of ‘integration’ is its implicit bias in favour of the dominant culture.”*) and Multicultural Skills (Items 41-60, e.g., *“In general, how would you rate your skill level in terms of being able to provide appropriate counselling services to culturally different clients?”*). Good psychometric properties have been reported, with Cronbach *alpha* of .75, .90 and .96 for Multicultural Awareness, Knowledge, and Skills respectively (D’Andrea et al., 1991), low inter-correlations between the subscales (D’Andrea et al., 1991), and factor analysis confirming the three-scale structure (Guy-Walls, 2007). Finally, a Wilcoxon matched pairs test for comparison of pre- and post-test results has confirmed internal consistency and construct validity (Ponterotto, Rieger, Barrett, & Sparks, 1994).

Minor adaptations were made to the MAKSS for cross-cultural application. Explicit reference to the United States in Items 35, 37 and 38 were replaced with references to the Netherlands, and reference to “Europe and Canada” were replaced by two countries which were part of the broader research: Australia and Singapore (Geerlings, Thompson, & Tan, 2017; Geerlings, Thompson, Bouma, & Hawkins, in press). The term “white” removed from the phrase “white mainstream clients” and the terms “gay men” and “gay women” were replaced with the phrases “men who are sexually attracted to men” and “women who are sexually attracted to women” respectively.

##### Marlow Crowne Social Desirability Scale Short Form (MCSDS-SF)

The short form of the MCSDS (Reynolds, 1982) contains 13 short statements (e.g., *“I’m always willing to admit it when I make a mistake”*) with a *true* or *false* forced-choice response format. A Kuder-Richardson-20 reliability of .76 and a correlation of .93 with the full MCSDS has been reported for the MCSDS-SF. It is recommended as a viable short form for measuring social desirability in response tendencies (Reynolds, 1982).

#### Procedure

After institutional ethics clearance, students, academic staff teaching into clinical psychology master’s programs, and alumni of these programs were sent an email invitation by the program director, or senior staff members.^i^ Emails were standardised and included a link to the online questionnaire hosted on Qualtrics survey software. As the recruitment emails were sent to program directors and senior staff members who forwarded the invitation to students, academic staff members and alumni, the response rate cannot be estimated. Data were exported into IBM SPSS for analysis.

#### Statistical Analysis

After data screening and assumptions testing, correlational analysis was conducted to identify collinearity between the MAKSS scales. Additional correlational analysis and MANOVA were used identify covariates for hypothesis testing, such as social desirability. Likewise, MCC differences between groups (students, academics, alumni) and universities were analysed to assess covariates. Two MANOVAs were conducted with the MAKSS subscales as DVs and group and university respectively as IVs.

A correlational analysis tested the first hypothesis that satisfaction with cultural training (a continuous variable) was associated with MCC. To test the second and third hypotheses that multicultural experience and minority identification were associated with MCC, a one-way between-subjects MANOVAs was conducted with multicultural experience and minority identification as IVs and the MAKSS scales as DVs, with social desirability and age as covariates.

### Results

#### Data Screening and Assumptions

Maximum likelihood estimation was used to manage occasional missing values in the MAKSS and MCSDS-SF of 27 participants. This method uses all available data to identify the parameter values that have the highest probability of producing the sample data. It is preferred over single imputation methods because it requires less stringent assumptions and provides a relatively unbiased solution to missing data (Baraldi & Enders, 2010). Missing demographic data were deleted list-wise.

The MAKSS and MCSDS-SF scores were normally distributed. The MAKSS *Awareness* and *Skills* scales and the MCSDS-SF had equal variances for the two universities, *F*(1, 105) = .05, *p* = .829; *F*(1, 105) = 1.77, *p* = .187*; F*(1, 105) = .30, *p* = .586, respectively, however, the MAKSS *Skills* scale had significant greater variability for university A, *F*(1, 105) = 4.63, *p* = .034. The MAKSS *Awareness*, *Knowledge* and *Skills* scales and the MCSDS-SF had equal variances for the groups of students, academics and alumni, *F*(2, 104) = .28, *p* = .756*; F*(2, 104) = .65, *p* = .524*; F*(2, 104) = .16, *p* = .854*; F*(2, 104) = .59, *p* = .558, respectively.

The reliability of the subscales of the MAKSS and the MCSDS-SF was acceptable, with Cronbach’s *alpha* of .74 for *Knowledge*, .88 for *Skills*, and .72 for social desirability. Cronbach’s *alpha* for *Awareness* was low at .52. Lower *alpha* for MAKSS Awareness is a known issue (D’Andrea et al., 1991), so the scale was used in the analysis.

Correlations between the variables were calculated to assess collinearity and are listed in Table 2. The MAKSS subscales were significantly inter-correlated, suggesting the MAKSS subscales each measured a related but distinct aspect of the MCC model. The correlation between *Skills* and *Knowledge* was quite high at *r* = .87, however, as highly inter-correlated DVs do not necessarily cause a loss of power in MANOVA (Cole, Maxwell, Arvey, & Salas, 1994), all three MAKSS scales were analysed.

#### Covariates and Group Differences

Social desirability and age were correlated with MCC to identify covariates. Social desirability significantly correlated with Skills, *r* = .38, *p* < .001, and was thus identified as a covariate for further analysis of the MAKSS. Age also significantly correlated with *Awareness*, *r* = .20, *p* = .035, but not with *Knowledge* or *Skills* (see Table 2). A one-way ANOVA with group (students, academics, alumni) as between-subjects IV and age as DV indicated significant differences between groups, Tamhane’s *T2 F*(2, 104) = 9.989, *p* < .001. Tamhane’s *T2* was used to adjust for unequal variances (indicated by a significant Levene’s Test, *p* < .001). Post-hoc tests suggested significant differences between all three groups: academics were significantly older (*M* = 43.00, *SD* = 10.708) than students (*M* = 25.09, *SD* = 3.766, *p* < .001) and alumni (*M* = 33.49, *SD* = 12.230, *p* = .030), and alumni were significantly older than students (*p =* .006). Therefore, both age and social desirability were covariates for subsequent analysis of the MAKSS.

**Table 2:** Correlations Between the Study Variables (n = 106)

Variable	Age	Satisfaction	Awareness	Knowledge	Skills
Satisfaction	-.15	–			
Awareness	.20*	-.09	–		
Knowledge	.18	.03	.31**	–	
Skills	.05	.03	.27**	.59**	–
MCSDS-SF	.06	-.01	-.04	.15	.38**

*Note*. Satisfaction: satisfaction with cultural elements in clinical psychology training; Awareness: MAKSSS Awareness scale; Knowledge: MAKSS Knowledge scale; Skills: MAKSS Skills scale; MCSDS-SF: Social desirability on MCSD-SF.

*p < .005. **p < .001.

Table 3 shows variability in means for the MCC subscales for groups of students, academics and alumni. A one-way MANOVA with group (students, academics, alumni) as between-groups IV and the MAKSS scales (Awareness, Knowledge, Skills) as DVs was conducted. Using Pillai’s Trace, no significant differences between groups of students, academics and alumni on MAKSS subscales were detected, *F*(6, 206) = 1.78, *p* = .105, so groups were combined in further analyses.

**Table 3:** Means and Standard Deviations for Variables (n = 106)

Variable	Students (*n* = 22)		Academics (*n* = 10)		Alumni (*n* = 74)	
	*M*	*SD*	*M*	*SD*	*M*	*SD*
MAKSS Awareness	2.53	4.39	2.58	3.27	2.62	4.06
MAKSS Knowledge	2.44	5.11	2.36	6.23	2.57	5.54
MAKSS Skills	2.58	6.82	2.40	6.06	2.70	7.20
MCSDS Social Desirability	0.46	2.62	0.56	3.23	0.52	3.10

*Note.* MAKSS scales score range: 1 – 4; MCSDS score range: 0 – 1.

A one-way MANOVA with respondents’ university as between-groups IV of and the MAKSS scales (Awareness, Knowledge, Skills) as DVs was conducted. Using Pillai’s trace, no significant differences between universities in MAKSS subscales were detected, *F*(3, 103) = 2.20, *p* = .092, so the two universities were combined in further analyses.

#### Hypothesis Testing

H1: A correlational analysis tested the hypothesis that satisfaction with cultural training (a continuous variable) was associated with MCC. Associations between satisfaction with cultural training and the MAKSS scales (Awareness, Knowledge, Skills) were not significant (all *p*s > .05; see Table 2).

H2 and H3: A one-way MANOVA with multicultural experience and minority identification as between-subjects IVs and the MAKSS scales (Awareness, Knowledge, Skills) as DVs, and with social desirability and age as covariates, tested the two hypotheses that multicultural experience and minority identification were associated with MCC. The variable for minority identification was constructed based on the self-reported cultural backgrounds; participants who identified as *autochtoon* were categorised as a cultural majority. The MANOVA suggested that while controlling for the effects of social desirability and age, cross-cultural experience had a significant main effect on the MAKSS subscales, *F*(3, 99) = 3.52, *p* = .018, minority identification was non-significant, *F*(3, 99) = 2.59, *p* = .057. Univariate ANOVAs detected a significant main effect of cross-cultural experience on MAKSS *Knowledge*, *F*(1, 101) = 4.00, *p* = .048, and on *Skills*, *F*(1, 102) = 9.66, *p* = .002, but not on *Awareness*, *F*(1, 101) = 0.00, *p* = .986. No interaction effects were identified, *F*(3, 99) = 1.26, *p* = .291.

### Discussion

It was hypothesized that satisfaction with cultural training, cross-cultural experience, and being a cultural minority would be associated with MCC. However, only cross-cultural experience had a significant effect on multicultural Knowledge and Skills, which is consistent with previous findings by Sehgal et al. (2011). In addition, in the exploratory studies from Hansen et al. (2006) and Allison et al. (1994), psychologists reported that cross-cultural experiences were most valuable in their cultural competency development. Study 1 confirms these findings using a standardised measure of cultural competency.

The results highlight the importance of personal experiences with diversity for cultural competency. The present findings are consistent with Allport’s (1954) influential *Contact Hypothesis* as well as more recent literature (e.g., Binder et al., 2009; Hewstone & Swart, 2011; Lemmer & Wagner, 2015; Pettigrew & Tropp, 2008), which affirms that inter-group contact reduces prejudice between majority and minority group members. A recently conducted meta-analysis of 515 studies demonstrated that inter-group cultural contact decreases prejudice through enhanced knowledge, reduced anxiety about inter-group contact, and increased empathy (Pettigrew & Tropp, 2008). These reported pathways from inter-group contact to reduced prejudice are at close parity with Sue et al.’s (1982) concepts of multicultural knowledge and skills. MCC research reported enhanced cultural knowledge (Sammons & Speight, 2008), increased cultural empathy (Burkard & Knox, 2004; Chao et al., 2011), and decreased racial prejudice (Castillo et al., 2007; Chao et al., 2011) as pathways to cultural competency. In other words, the present findings are which is consistent with literature on intergroup contact and point out to the importance of cross-cultural contact for cultural competency development.

In contrast to previous findings (D’Andrea et al., 1991; Tummala-Narra et al., 2012), Study 1 suggests that cultural competency is not dependent on the way students, academics and alumni perceive their graduate education. This inconsistency with previous findings may be related to the type of cultural training received. D’Andrea et al. (1991) only detected an effect on cultural competency for tailored multicultural training in graduate programs. Such tailored training was absent in the universities participating in Study 1, possibly explaining the inconsistent result. In addition, Tummala-Narra et al.’s (2012) survey assessed the effects of multicultural training undertaken after formal education. As psychologists participate in specific multicultural workshops that suit their needs as professionals, it is likely that such tailored training had a more profound effect on cultural competency than standardised cultural training provided to students in postgraduate programs. Consequently, the potential benefits of tailoring cultural training to students’ or professionals’ specific cultural competency needs could be explored in future research.

Finally, Study 1 shows that cultural minority and cultural majority individuals are equally culturally competent. This result is inconsistent with American research, which reported higher self-reported cultural competency among students of colour (Pope-Davis et al., 1995). Pope-Davis et al. (1995) considered heightened cultural competency of minority students a result of their “different experiences” (p. 327). However, while the United States and the Netherlands both have a diverse population consisting of a ‘white’ majority with various minority groups, it cannot necessarily be assumed that American and Dutch cultural minority individuals have similar experiences. This was reflected in the survey responses in Study 1, which revealed that 41% of participants disagreed with the statement that *“minority experiences in the Netherlands are similar to experiences of minorities in neighboring countries*.*”* Thus, it is important to gain insight into the actual experiences of clinical psychology training and practice in the Netherlands. Study 2 was designed to address this.

## Study 2: Interviews on Multicultural Practice

### Method

#### Participants

Fourteen people were interviewed, including 5 students (age: *M* = 26.4, age range: 25 – 34 years), 5 academics (age *M* = 50, age range: 31 – 66 years, one academic did not report age), and 4 alumni of a clinical psychology program (age: *M* = 45, age range: 27 – 58 years). Eight participants were affiliated with University A, six with University B. Eight participants identified as *autochtoon* (cultural majority) Dutch, a further three as a cultural minority, and another three as a foreigner. Further demographic information was collected but not reported in order to maintain confidentiality.

#### Procedure

After institutional ethics clearing, students and academics of clinical psychology in two universities were invited to participate via the online survey of Study 1 (*n* = 13), and through snowball sampling (*n* = 1). All semi-structured interviews were conducted face-to-face at the participants’ universities or workplaces, or in public places nominated by the participant. All interviews were conducted in English by the same experienced interviewer for an average duration of 30 minutes (range: 20 – 45 min). Interviews were audio-taped and were transcribed verbatim with NVivo software. Participants were invited to check the transcripts for accuracy and sufficient de-identification; five participants provided feedback, including correction of minor grammatical errors and additional information related to the interview.

An interview outline of open-ended questions was designed to invite participants to describe and reflect upon their experiences of studying (e.g. *“How much consideration for cultural competency is included in the program?”*), teaching (e.g. *“How do you prepare students for culturally competent practice?”*), or practicing clinical psychology (e.g. *“Can you tell me about a multicultural clinical practice situation you encountered?”*). The outline was piloted with one clinical psychologist not otherwise involved in the study, and minor modifications were made. The outline was used in a flexible manner and interviewees were explicitly invited to introduce topics or ideas related to the research topic.

#### Data Analysis

Qualitative analysis used an Interpretative Phenomenological Analysis framework to gain insights into the lived experiences of events from the perspective of the participant (Larkin, Watts, & Clifton, 2006; Smith, Flowers, & Larkin, 2009). This approach allowed for discovery of new themes and constructs based on actual experiences, as opposed to the confirmatory approach used in Study 1. To ensure validity and transparency of the analysis, one analysis was audited by a participant and a second by another researcher. While a lack of clear criteria for IPA should be acknowledged, both auditors considered the researcher’s analysis highly plausible and sufficiently similar to their own interpretations of the transcripts, so it was decided that no further auditing was required.

After data familiarization, descriptive, linguistic and conceptual comments on the transcripts were noted. Based on these comments, themes were constructed that focused on the participant’s experiences. For each of the 14 transcripts, these themes were clustered based on similarity in meaning, with each group representing a superordinate theme. To form homogenous samples for IPA, student, academic and alumni interviews were then analysed in three separate groups. Group analyses focused on identifying patterns and constructing master themes that summarised the experiences of each group (students, academics, alumni). However, as demonstrated in Table 4, the themes showed considerable overlap between the groups. Therefore, superordinate themes were renamed for consistency between the groups and collated into shared master themes. Superordinate themes were included in master themes if they were present in at least half of the interviews in student, academic, or alumni interviews (Smith et al., 2009).

**Table 4:** Distribution of Master and Superordinate Themes per Participant Group

Theme	Students					Academics					Alumni			
	S1	S2	S3	S4	S5	AC1	AC2	AC3	AC4	AC5	AL1	AL2	AL3	AL4
Limitations of clinical psychology														
a. Western bias	X	X	X	X	X			X			X	X	X	X
b. Rigidness of science						X	X	X	X	X	X	X	X	X
c. Limits of training	X	X	X	X	X	X	X	X	X	X	X	X	X	X
Strategies for culturally competent practice														
a. Cross-cultural communication	X	X	X	X			X	X	X	X		X	X	X
b. Professional relationship	X											X	X	X
c. Person-centred practice			X			X	X		X	X		X	X	X
Teaching and learning cultural competency														
a. Cross-cultural experience	X	X	X	X	X	X		X	X	X	X	X	X	X
b. Clinical psychology curricula	X	X	X	X	X				X		X		X	X
c. Life experience	X	X	X	X		X	X		X		X	X	X	X

### Results

The master themes cover student, academic and alumni experiences of the limitations of clinical psychology, strategies for culturally competent practice, and strategies for teaching and learning cultural competency. The themes are discussed below for students, academics, and alumni together. Participant quotes are de-identified and italicized, and brackets are used to identify material that was omitted, added, or changed in the participant’s quotes for clarification and confidentiality.

#### Limitations of Clinical Psychology

All participants experienced limitations of clinical psychology in cross-cultural application, including a ‘western’ bias, rigidness of psychological science, and limits to training.

##### Western bias

Students and alumni experienced clinical psychology as culturally biased: *“The main root of psychology is western-orientated.”* In their view, some of the western ‘roots’ of psychology were American behaviourism, Cartesian mind-body dualism, and psychoanalysis, provocatively summarized as the knowledge of *“old white men with beards.”* These western origins have resulted in models and practices that are tailored toward working with clients who are highly educated, and who value individualism, atheism, and *“rationalism”*. Frequently stated examples of experiences of these values are the aim of self-actualisation and the practice of discussing and analysing emotions in therapy. According to participants, culturally competent practitioners are aware of these biases.

##### Rigidness of science

Academics and alumni experienced psychological science as insufficiently flexible for application in cross-cultural application. Psychological science, influenced by biomedicine, is analytical rather than holistic. Consequently, the resulting psychological knowledge and models neglect cultural and personal variation and are therefore not always useful to inform clinical practice: *“If you truly treat patients, broad numbers and studies mean nothing. They are just a framework for you to treat the individual. The big groups* […] *tell you nothing about the individual.”*

Identifying as scientist-practitioners, academics and alumni felt that in order to use different, more culturally appropriate approaches in training or practice *“you need a theoretical and research-driven framework. And I am not aware of any such things.”* Participants experienced a shortage of research into cultural issues, causing a lack of knowledge and evidence-based practices to aid cultural competency. Consequently, according to academics and alumni, an important cultural competency was the ability to apply generic, scientific knowledge while paying attention to client-specific circumstances.

##### Limits to training

A third and final factor limiting clinical psychology’s cross-cultural application was clinical psychology’s organisation into training programs. All participants experienced clinical psychology master’s degrees to be insufficient preparation for culturally competent practice. Alumni recalled the difficulty of starting to practice cross-culturally: *“I felt like I was walking on eggs because my whole training and outlook didn't match the outlook or the way these* [culturally different] *people were oriented towards their worlds.”* In addition, participants expressed concern about how culturally unaware psychology master’s graduates are:

*I'm very heavily trained in one view, and while I believe it is a good view, I am not naive enough to believe it's the* only *view.* […] *But I fear… I don't think most psychologists fully realise it. And I don't blame them, because how can they? They've never been taught...*

Academics acknowledged the need to pay more attention to cultural competency in master’s programs but felt that their hands were tied: *“I think there isn’t really room for much anymore additions in the master because it’s only one year.”* Consequently, cultural training was not a structural part of the curriculum, and some academics expressed concern about their ability to meet the needs of the culturally diverse client and student populations: *“I hope that there is intermingling between the international students and the Dutch students. And I hope that that way students can* [at least] *learn something about cultural differences.”* The structure of clinical psychology training thus limits cultural competency development.

#### Strategies for Culturally Competent Practice

Participants identified three strategies for culturally competent practice: cross-cultural communication, building a good professional relationship, and person-centred practice. All three competencies are generally applicable.

##### Cross-cultural communication

The ability to effectively communicate cross-culturally was identified by students, academics and alumni as an important cultural competency for practice and training. Communication strategies were generally among the first cultural competencies participants introduced into the interviews, for example:

*I slowed my speech tempo in order to ensure that we were really understanding each other.* […] *And if a person has a certain non-verbal way of expression I enquire about what that means and try to elicit their stories as to what is going on. Less taking for granted that you really understand each other without saying, as you might do with someone from your own culture.*

Related cross-cultural communication competencies included recognising and adjusting to different communication styles and norms, identifying and alleviating communication and expression difficulties, effectively communicating verbally and non-verbally, and ensuring mutual understanding.

##### Professional relationship

In addition to cross-cultural communication, alumni identified the ability to establish a professional cross-cultural relationship as an important cultural competency. In cross-cultural practice, alumni reported to *“take things more slowly and take more time to establish a personal relationship and building trust.”* In order to build trust and set up the professional relationship carefully, alumni reported to openly display empathy and care for their clients, and to take more time to get to know one another. The professional relationship was a key strategy in cross-cultural practice: *“I have to make sure that my client is really motivated and really wants and believes in me. I adapt a bit to their culture and so they'll think: oh yes, this one will support me.”*

##### Person-centred practice

A third and final cultural competency, identified by academics and alumni, was person-centred practice. A generic therapeutic skill, person-centred practice involves trying to understand each client’s cultural and social circumstances as well as expressions of individuality and adjust the therapeutic approach to each unique individual. The identification of person-centred practice as a competency for multicultural practice de-emphasises culture and values the individual client:

*There is always this thing: do you really need to have cultural information or do you always need to adapt to your patients? And that is very fundamental question.* […] *I think the most important thing is to be able to sit next to your patient. To understand how they see things, how they experience it.*

#### Strategies for cultural competency development

All participants identified the need for cultural competency. Three important experiences for developing cultural competency were revealed: cross-cultural social relations, clinical psychology curricula, and generic life experiences.

##### Social relations

Students, academics and alumni considered their cross-cultural social relations as essential in their cultural competency development. Every participant recalled experiences of cross-cultural intimate relationships or friendships, or cross-cultural interactions with colleagues, clients, or neighbours, or strangers, or a combination of these experiences. These have been key in participants’ cultural self-reflection, insights into other cultures, and generic feeling of cross-cultural competency. Relatedly, some cultural minority participants felt that they had an advantage over cultural majority psychologists through increased understanding of cultural differences. This highlights the value of cross-cultural experiential activities for cultural competency: *“If you experience for yourself that your basic assumptions are not* the *assumptions, then that experience may be worth it.”*

##### Clinical psychology curricula

Participants expressed the need for more cultural training opportunities. Students and alumni opined that cultural training should be part of standardised clinical psychology curricula, to create a baseline exposure to cultural issues. Establishing a baseline is especially pertinent as, despite diversification, most clinical psychology students, academics, and practitioners are still *“white”* and belong to the cultural majority group in the Netherlands. They may be rarely exposed to cultural issues and unaware of the importance of culture: *“What I see is the problem with my fellow students, because they didn’t really know how important* [the cultural] *background is for the client.”*

Formal cultural training could thus raise cultural awareness, and also increase cultural knowledge. Students and alumni experienced positive effects of having background cultural knowledge on their clients: *“You have to know about their culture and religion in order for you to understand what their* [mental illness] *means to them and how it… how it expresses itself.”* Consequently, the integration of cultural issues into master’s programs could aid culturally competent practice through enhancing cultural awareness and knowledge.

##### Life experience

Finally, students, academics, and alumni felt that having sufficient life experience is a prerequisite for cultural competency development. This recurrent theme in the interviews can be summarized as follows: *“Your question was, how do I... how did I learn* [cultural competency]? *I think it is based on experience…”* As ‘experience’, some participants recalled their many years of clinical practice, others emphasised the importance of working in different environments, while others recalled their experiences of travelling or living abroad, or even life events such as marriage and having children. These experiences all signal that emotional maturity could be a precondition for cultural competency development. This implies that it may be difficult to ‘teach’ cultural competency to young, inexperienced students, which was reflected in interviews with academics: *“Well some of these students, they* […] *come in age twenty-one... live with their parents… and may not be ready yet.”*

### Discussion

Study 2 was designed to explore how students, academics, and alumni of clinical psychology experience preparation for culturally competent clinical psychology practice. The results show that despite the efforts to increase diversity in the sector, clinical psychology training and practice still does not cater for cultural diversity in the Netherlands. Study 2 indicates the ‘western’ cultural bias and ethnocentrism of the models and practices as one reason. In addition, as reported previously (e.g., Henrich, Heine, & Norenzayan, 2010), psychological science continues to neglect diversity, leaving scientist-practitioners empty-handed with no information to guide their cultural training and practice. Meanwhile, training and practice has become more regulated and structured. Due to interplay of these factors, clinical psychology curricula do not reflect the increasing cultural diversity of the Dutch society and are delivering practitioners who feel ill-equipped for multicultural practice. The need for cultural inclusiveness in the Dutch mental health care sector has been acknowledged previously (e.g., Bekker & van Mens-Verhulst, 2008; Knipscheer et al., 2011; Van Dijk, 2008), and in Study 2 it was expressed by participants who completed their training two decades ago, as well as by current students, suggesting that updates in curricula are past due.

The reported strategies for multicultural practice indicate to possible directions for cultural competency training. The most pertinent cultural competency identified in Study 2 was the ability to effectively communicate cross-culturally. This contributes to the growing recognition of the role of communication in cross-cultural relations in the Netherlands (e.g., Schinkel, Schouten, & van Weert, 2010; Shadid, 2007; Shadid & Van Koningsveld, 1999; Universiteit Utrecht, 2016). Research has demonstrated that communication patterns differ when Dutch medical doctors interact with cultural minority or majority patients. Communication with cultural minorities was characterised by less empathy and involvement, and more emphasis on the patients’ symptoms (Meeuwesen, Harmsen, Bernsen, & Bruijnzeels, 2006). In clinical psychology practice, such patterns may interfere with forming a therapeutic alliance. Thus, intercultural communication training may benefit cross-cultural practice. Indeed, health care practitioners who were competent cross-cultural communicators expressed more empathy (Gibson & Zhong, 2005), were more culturally sensitive, and felt more culturally competent (Ulrey & Amason, 2001). Thus, the potential benefits of training clinical psychologists in cross-cultural communication merit further research enquiry.

The generic applicability of the cultural competencies identified in Study 2 suggests that a model for cultural competency may help guide training efforts. The competencies reported in Study 2 are not specific to cultural groups, which is important for preventing cultural stereotyping (Epner & Baile, 2012; Kleinman & Benson, 2006), and may assist multicultural practice through adaptations tailored to each individual client. Similarly, the competencies of multicultural *awareness*, *knowledge* and *skills*, described in Sue et al.’s (1982) MCC, are generally applicable across cultural groups. In Study 2, Sue et al.’s (1982) multicultural awareness and knowledge were reflected in the theme *clinical psychology curricula.* In addition, *cross-cultural communication* is one aspect of Sue et al.’s (1982) multicultural skills. However, the competency of building an effective cross-cultural *professional relationship*, also identified in Study 2, is not reflected in Sue et al.’s (1982) MCC. This competency resembles *multicultural counselling relationship,* described by Sodowsky et al. (1994) as openness and warmth expressed toward culturally different clients. More research is required to assess which standardised model for cultural competency is most suitable, or, perhaps, if a new model of cultural competency should be developed for to guide cultural competency training in the Netherlands.

Study 2 suggests that culturally competent practitioners are generally therapeutically competent, are emotionally mature due to myriad professional and life experiences, and are familiar with diversity. The importance of personal familiarity with diversity for cultural competency development is consistent with Study 1 and existing literature (e.g., Allport, 1954; Binder et al., 2009; Hewstone & Swart, 2011; Lemmer & Wagner, 2015; Pettigrew & Tropp, 2008). Cross-cultural contact may enhance cultural awareness and knowledge, and foster a sense of cultural competency. However, the need to integrate cultural components in master’s curricula, pointed out by students and alumni, suggests that commencing clinical psychologists may need more preparation for cultural competency. These practitioners miss the professional experience, and often also the life experience needed to feel culturally competent, as they are generally younger (Study 1). This suggests that, apart from integrating cultural training into clinical psychology curricula, it may be beneficial to encourage cross-cultural social encounters between cultural majority and minority students in the classroom, or to facilitate study abroad programs to increase students’ experiences with diversity.

All interview participants were familiar with cultural issues in clinical psychology. This suggests the possibility of self-selection bias: those with strongest opinions may be more likely to volunteer to be interviewed. Thus, the findings may not be fully representative of the population of clinical psychology students, academics and alumni in the Netherlands. Consequently, the importance of culture in clinical psychology training and practice may be overestimated. More research is needed to assess the validity of the present findings in other universities and regions of the Netherlands.

## Conclusion

This is, to our knowledge, the first mixed-methods study of culturally competent clinical psychology practice in the Netherlands using the theory of MCC. Study 1 and 2 demonstrate the importance of experiences with diversity for cultural competency, as well life experience and cultural training. While the standardised model of MCC may be partly applicable, more research is needed to determine which model for cultural competency is best suited to guide cultural competency training in the Netherlands. The present paper has made suggestions for further research that may assist in this enquiry. Such research is timely. The experiences of students, academics and alumni indicate that cultural competency continues to need attention in research and practice, so that psychologists feel confident and well-prepared to cater for the mental health needs in the increasingly diverse society.
